# Analytical methods used in estimating the prevalence of HIV/AIDS from demographic and cross-sectional surveys with missing data: a systematic review

**DOI:** 10.1186/s12874-020-00944-w

**Published:** 2020-03-14

**Authors:** Neema R. Mosha, Omololu S. Aluko, Jim Todd, Rhoderick Machekano, Taryn Young

**Affiliations:** 1 Division of Epidemiology and Biostatistics, Faculty of Medicine and Health Sciences, Stellenbosch University, P.O. Box 241, Francie van Zijl Drive, 7505 Tygerberg, Cape Town, South Africa; 2 Mwanza Intervention Trials Unit, P.O. Box 11936, Isamilo road, Mwanza, Tanzania; 3 National Institute for Medical Research, Mwanza Centre, P.O. Box 1462, Isamilo road, Mwanza, Tanzania; 4 London School of Hygiene and Tropical Medicine, Keppel St, Bloomsbury, London, WC1E 7HT UK

**Keywords:** Missing data, Non-response, Surveys, HIV/AIDS

## Abstract

**Background:**

Sero- prevalence studies often have a problem of missing data. Few studies report the proportion of missing data and even fewer describe the methods used to adjust the results for missing data. The objective of this review was to determine the analytical methods used for analysis in HIV surveys with missing data.

**Methods:**

We searched for population, demographic and cross-sectional surveys of HIV published from January 2000 to April 2018 in Pub Med/Medline, Web of Science core collection, Latin American and Caribbean Sciences Literature, Africa-Wide Information and Scopus, and by reviewing references of included articles. All potential abstracts were imported into Covidence and abstracts screened by two independent reviewers using pre-specified criteria. Disagreements were resolved through discussion. A piloted data extraction tool was used to extract data and assess the risk of bias of the eligible studies. Data were analysed through a quantitative approach; variables were presented and summarised using figures and tables.

**Results:**

A total of 3426 citations where identified, 194 duplicates removed, 3232 screened and 69 full articles were obtained. Twenty-four studies were included. The response rate for an HIV test of the included studies ranged from 32 to 96% with the major reason for the missing data being refusal to consent for an HIV test. Complete case analysis was the primary method of analysis used, multiple imputations 11(46%) was the most advanced method used, followed by the Heckman’s selection model 9(38%). Single Imputation and Instrumental variables method were used in only two studies each, with 13(54%) other different methods used in several studies. Forty-two percent of the studies applied more than two methods in the analysis, with a maximum of 4 methods per study. Only 6(25%) studies conducted a sensitivity analysis, while 11(46%) studies had a significant change of estimates after adjusting for missing data.

**Conclusion:**

Missing data in survey studies is still a problem in disease estimation. Our review outlined a number of methods that can be used to adjust for missing data on HIV studies; however, more information and awareness are needed to allow informed choices on which method to be applied for the estimates to be more reliable and representative.

## Background

Worldwide, the HIV/AIDS epidemic is still a problem. It is estimated that currently, 37million people are living with HIV (PLHIV), with 70% of these in sub-Saharan Africa [1]. The estimated HIV prevalence is usually obtained from nationally representative, population studies such as demographic health surveys (DHS). However, surveys often have a problem of missing data, which can be a source of bias and can reduce study precision [2].

Accurate HIV prevalence estimates are important for monitoring and evaluating the ongoing programs, for the prevention and treatment of HIV and the allocation of resources within countries [3]. The available literature and guidelines on reporting observational studies(STROBE) suggest that for results to be efficient, the amount of data missing and methods used for handling the problem must be reported [4, 5]. The STROBE guidelines go further and explain the importance of reporting the reasons for missingness, which may include unit non-response, where a study participant or household are missing from the entire study, or item non-response, where some questions are not responded to, or wrongly entered in the database. The common reason for missing data in HIV studies includes the refusal to test or non-response to the survey [3, 6]. However, few studies report the proportion of missing data or even fewer describes the methods used to adjust for missing data [7].

Most of the published articles for estimating the prevalence and incidence of any diseases are based only on the use of complete case data analysis or available case analysis [8]. A few of the articles describe ad hoc methods such as the use of dummy variable and mean imputation for the estimation of disease prevalence and incidence. And even fewer articles describe more advanced methods for adjusting for missing data, such as inverse probability weighting, instrumental variables and multiple imputations [7, 9].

Many demographic and cross-sectional surveys have been conducted to estimate HIV prevalence and have been reported in peer-reviewed journals, but few recognise the bias that could be present from missing data. Editors and authors need to consider how these estimates have been obtained and how missing data have been addressed. It is important that advanced methods to adjust for missing data are incorporated in the analysis of HIV survey data to reduce the bias in the estimates. Failure to adjust for missing data may result in biased estimates of parameters of interest and can have a negative impact on controlling the epidemic [9]..

This study aimed to conduct a review of articles from HIV surveys with missing data to determine what analytical methods or techniques have been used during, estimating HIV prevalence. Also, to identify the methods used for sensitivity analysis to assess the robustness of the assumptions used.

## Methods

Two guidelines were used during the conducting and reporting this review, the Preferred Reporting Items for Systematic Reviews and Meta-Analyses (PRISMA) [10] and Strengthening the Reporting of Observational Studies in Epidemiology (STROBE) [5].

### Eligibility criteria and search strategy

An information specialist searched five different databases on 13th August 2018. The database list included Medline via PubMed, Web of Science Core Collection, Latin American and Caribbean Sciences Literature, Africa-Wide Information and Scopus. (Additional file 1).

Studies published from population surveys, either demographic or cross-sectional studies from January 2000 to August 2018 on estimating the prevalence of HIV/AIDS written in English were eligible to be included in the review. All articles had to include a statement or paragraph on how missing data or non-response was handled during analysis in the abstract.

### Study selection procedure

All potential studies were imported into Covidence screened for their titles and abstracts to identify the relevant studies (Covidence systematic review software, Veritas Health Innovation, Melbourne, Australia. Available at www.covidence.org). Two independent reviewers applied the pre-specified criteria to select abstracts and to reject abstracts that are not relevant, with a third reviewer acting as a tiebreaker. Full text of all selected abstracts were obtained and assessed against the eligibility criteria. Disagreements were resolved through discussion between the two reviewers and the third reviewer.

### Data extraction and risk of bias assessment

Before data extraction, all studies were assessed for the possibility of bias using a tool adapted from Hoy et al. .2012 [7, 11]. The Hoy tool has been designed to assess the risk of bias in population-based prevalence studies; it comprises of 10 domains which allow us to identify the study included if it has a low or high risk of bias. The items include a question that assessed the internal validity on the representativeness of the national or target population, sampling strategy used, the likelihood of non-response and question that assessed the external validity on how data were collected and analysed, reliability and validity of the estimates(Additional file 2). We used Kappa statistics to assess the agreement between the two reviewers on the full text studies included. The values where set as ranges of 0 to 0.20 as slight agreement; 0.21 to 0.40, fair agreement; 0.41 to 0.60, moderate agreement; 0.61 to 0.80, substantial agreement; and greater than 0.80 almost perfect agreement.

A piloted data extraction form with structured questions was used to collect data from the included studies independently by the two reviewers. We collected data on year of publication, place of study, type of study, sample size and if adjusted for missing data, how the outcome of interest was analysed, primary analysis and methods used to adjust for missing values. Discrepancies were discussed and resolved; an external reviewer was invited in if the consensus was not achieved from the two reviewers. The data extraction tool used is included as Additional file 3.

### Data analysis

The extracted data were analysed through a quantitative approach. All the variables collected were described and summarised using flow chart and tables. The characteristics of individual studies included were described. Proportions of studies that reported missing values and the methods used to adjust for missing data or selection bias were summarised in the following way. Methods used for analysis were also described and, any other studies that performed sensitivity analyses for any of the methods were also quantified.

## Results

**Fig. 1:**
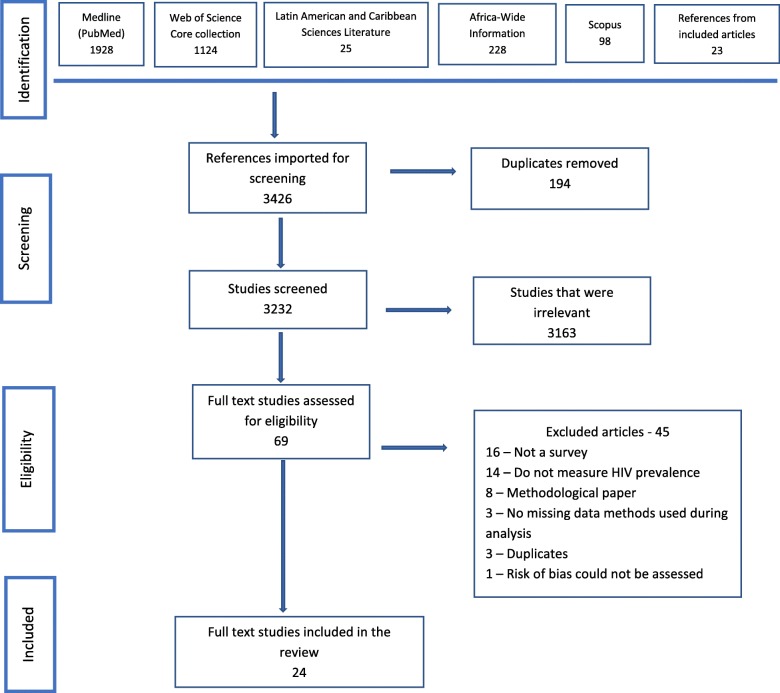
A PRISMA flow diagram on the search and selection of studies process

A total of 3426 citations were identified, 194 duplicates removed, 3232 screened, and 69 full articles obtained. The excluded abstracts were not surveys, or were not estimating HIV prevalence, or did not include any missing data methods to estimate HIV. Following full-text eligibility assessment, 24 studies were included while 45 studies were excluded due to not being a survey [12], not measuring HIV prevalence [13], being a methodological study [8], having no missing data methods used during analysis [3], duplicates [3] and 1 study where we could not assess the risk of bias, as it did not show the adjusted HIV prevalence after using the advanced methods for missing data. Table 1 shows the details of the excluded studies and a flow chart of the systematic review is provided in Fig. 1.

**Table 1:** Excluded studies and reasons for exclusion

Reference	Reason for exclusion	n (%)
Arpino 2014, Barbosa 2002, Blum 2010, Dagne 2015, DiRienzo 2009, Guan 2017, Huang 2012, Kenward 2001, Nyirenda 2010, Obare 2010, Patrician 2002, Scharfstein 2003, Sun 2018, Tian 2007 [12–24].	Not a survey	14 (31.1)
Bärnighausen 2012, Grassly 2004, Hlalele 2008, Kranzer 2008, Liu Y 2015, Liu S 2015, Mistry 2008, Nelwamondo 2007, Pantanowitz 2009a, Pantanowitz 2009b, Rosinska 2013, Schomaker 2018, Shah 2014, Westreich 2012, Wirth 2010, Wu 2001 [25–39].	Do not measure HIV prevalence	16 (35.6)
Boerma 2003, Brookmeyer 2010, Clark 2012; Garcia-Calleja 2006, Gouws 2008, Hund 2013, Korenromp 2013, Larmarange 2014 [2, 6, 40–44].	Methodological article	8 (17.8)
Alkema 2008, Montana 2008, Kayibanda 2011 [45–47].	No missing data methods used in the analysis	3 (6.7)
McGovern 2015a, Obare 2010, Pentanowitz 2009a [23, 33, 48].	Duplicate	3 (6.7)
Ng 2013 [49].	Could not assess the risk of bias	1 (2.2)

### Description of included studies

Out of the24 studies, 12 (50%) were Demographic Health Survey (DHS) studies [48–60], Seven (29%) Cross-sectional surveys [52, 61–66], three (13%) population surveys [67–69] and 2(8%) a mixture of Demographic Health Survey and Aids Indicator surveys [50, 70]. These studies were published between 2006 to 2018, and more than 95% of the studies were done in sub-Saharan Africa. The age of the participants ranged from 12 to 64 years, with more women than men participants. Table 2 provides a summary of 10 of the included studies which used a single, unique source of data, and did not use DHS data.

**Table 2:** Description of included studies which used only one source of data

No	Study ID	Country	Year of survey	Year of publication	Sample size	Age of included participants	Type of study
1	Floyd [61]	Malawi	2006–2010	2013	17,000	≥15	Cross-sectional survey
2	Harling [71]	South Africa	2012	2017	42,357	≥15	Population Survey
3	Jessens [62]	Namibia	2008–2009	2014	1992	≥12	Cross-sectional survey
4	Kendall [63]	Angola	2011	2014	792	≥18	Cross-sectional survey
5	Kerr [65]	Brazil	2016	2018	4176	≥18	Cross-sectional survey
6	Kerr [64]	Brazil	2009	2013	3859	≥18	Cross-sectional survey
7	Leacy [68]	Zambia	2006–2010	2016	34,446	≥18	Population survey
8	McGovern [69]	South Africa	2009	2015	25,392	≥15	Population survey
9	Reiners [52]	Ethiopia	2003–2004	2009	1650	≥16	Cross-sectional survey
10	Ziraba [66]	Kenya	2006–2007	2010	4767	≥15	Cross-sectional survey

Fourteen studies had multiple sources of data that were analysed. Whereby in other studies datasets were used more than once. All these studies used DHS data from different countries in Sub-Saharan Africa. The most common data set used was from Zambia DHS (2007) and Zimbabwe DHS (2006). A study by Marino et al. used more datasets than any other study (28/32) followed by Hogan et al. (27/32) and Mirsha et al. (14/32). Table 3 shows the intersection of data usage from the 14 studies with multiple sources of datasets, including DHS data.

**Table 3:** Display of multiple datasets usage

Country	Year of survey	Author and Year of Publication													
		Hogan, 2012	Tchetgen, 2013	Reniers, 2009	Marden, 2018	Mara, 2017	McGovern, 2015a	McGovern, 2015b	Martson, 2008	Marino, 2018	Mishra, 2008	Clark, 2014	Barnighausen,2011	Mishra,2006	Chinomona,2015
Burkina faso	2003	**X**								**X**	**X**			**X**	
Cambodia	2005													**X**	
Cameroon	2004	**X**		**X**						**X**	**X**			**X**	
Congo Brazzaville	2009	**X**								**X**					
Congo DR	2007	**X**								**X**					
Cote dívoire	2005	**X**							**X**	**X**	**X**			**X**	
Ethiopia	2005	**X**							**X**	**X**	**X**			**X**	
Ghana	2003	**X**		**X**			**X**		**X**	**X**	**X**			**X**	
Guinea	2005	**X**								**X**					
India	2006													**X**	
Kenya	2003	**X**							**X**	**X**				**X**	
Kenya	2009	**X**							**X**	**X**	**X**				
Lesotho	2004	**X**		**X**					**X**	**X**	**X**	**X**		**X**	
Lesotho	2009								**X**	**X**		**X**			
Liberia	2007	**X**								**X**					
Malawi	2004	**X**		**X**					**X**	**X**	**X**			**X**	
Malawi	2010	**X**							**X**	**X**					
Mali	2001	**X**								**X**					
Mali	2016									**X**					
Mozambique	2009	**X**								**X**					
Niger	2006	**X**								**X**					
Rwanda	2005	**X**								**X**	**X**			**X**	
Senegal	2005	**X**		**X**						**X**					
Sierra Leone	2008	**X**								**X**					
Swaziland	2007	**X**				**X**				**X**		**X**			
Tanzania	2004	**X**								**X**	**X**			**X**	
Tanzania	2008	**X**								**X**					
Uganda	2005										**X**			**X**	
Zambia	2002	**X**								**X**					
Zambia	2007	**X**	**X**		**X**	**X**	**X**	**X**		**X**		**X**	**X**	**X**	
Zimbabwe	2006	**X**		**X**		**X**			**X**	**X**	**X**	**X**			
Zimbabwe	2011	**X**													**X**

**Legend**: **X**-Dataset used

### Risk of Bias assessment

The overall Cohen’s kappa coefficient statistic for the two authors screening all the included studies was estimated to be 0.93. We had a higher risk of bias on domains that assessed the internal validity of the studies compared to domains assessing external validity. Almost all studies had a higher risk of bias on Domain 4 which looked on likelihood of non-response (23/24), followed by Domain 1 which looked on the target population is a close representation of the national population (10/24) (Appendix 4). Only one study had a high risk of bias in terms of domains that looked on external validity (domain 8), which asked if the same mode of data collection was used for all subjects. Additional files 2 and 4 shows in detail all the domain assessed, and results of the assessment done.

### Characteristics of the missing data

Only 21 of the 24 studies reported the response rate for an HIV test. It ranged from 32 to 96%. All the studies gave a reason for the missing data reported, major reason being the participant refused to consent to an HIV test and 8 (33%) studies identified further missing data from unit-nonresponse Six (25%) studies reported missing data as a separate outcome, while only 9 (38%) had a result table comparing the participants with complete data and the ones with missing data. Table 4 provides a summary of the mentioned characteristics.

**Table 4:** Summary of the missing data characteristics (*n* = 24)

CHARACTERISTICS	n	%
**Response rate reported**		
Yes	21	88
No	3	22
**Response rate reported**		
< 70%	2	9
70–80%	10	48
> 80%	9	43
**Reasons for missing data reported**		
Yes	24	100
No	0	0
**What were the reasons reported**		
Refusal to test for HIV	24	100
Absence	3	13
Technical problems	1	4.2
**Type of missing data mentioned**		
Unit non-response	8	33
Unit and Item non-response	16	67
**Missing data reported as a separate outcome**		
Yes	6	25
No	18	75
**A summary table to compare participants with complete data vs incomplete data**		
Yes	9	38
No	15	62

### Analytical methods used in the analysis

All the 24 studies included in the analysis used complete case analysis method as their primary method of analysis. Multiple imputations 11(46%) was the most advanced method used to adjust for missing data followed by the Heckman’s selection model 9(38%). Single Imputation and Instrumental variables method were used in only two studies each, with 13(54%) other different methods used in several studies. Ten studies (42%) applied more than two methods in the analysis, with a maximum of 4 methods in two studies. Table 5 describes the methods used to adjust for missing data on estimating HIV prevalence.

**Table 5:** Missing data methods used in the analysis

CHARACTERISTICS	n	%
**Major methods used for analysis**		
Complete case analysis	24	100
Single imputation	2	8
Multiple Imputation	11	46
Instrumental variables	2	8
Heckman’s selection model	9	38
Other methods	13	54
**Other methods used**		
Age standardization	2	8
Upper bounds and lower bounds	1	4
Copulae models	2	8
Logistic prediction models	1	4
Refusal rate adjustment	1	4
Mobility rate adjustment	1	4
Random effect bias model	1	4
HIV self-report imputation	1	4
Prevalence ratio inflation factor	1	4
HIV risk ratio adjustment	1	4
Network imputation using recruitment chain	1	4
Conditional probability equations	1	4
**Maximum number of methods used per study**		
2	14	58
3	8	34
4	2	8

Only 1 study mentioned the pattern identified of the missing data, while more than half 13(53%) of the studies stated the mechanism assumed in the analysis. Of the 13 studies that mentioned the mechanism used during analysis, all studies assumed data to be MCAR for the complete analysis, 11 assumed data to be MNAR, ten assumed data to be MAR and seven studies assumed both MAR and MNAR. For the studies that used Multiple imputation method, only 3 (27%) stated the number of imputed data sets in the analysis, but seven (64%) mentioned the variables used in the imputation model. On assessing the robustness of the results only 6(25%) studies conducted a sensitivity analysis, while 11(46%) studies had a significant change of estimates after adjusting for missing data. Table 6 provides details on the different aspects of the analysis strategy and methods.

**Table 6:** Further information on the analysis and results conclusion provided

CHARACTERISTICS	n	%
**Missing data pattern stated in the analysis**		
Yes	1	4
No	23	96
**Missing data mechanism stated in the analysis**		
Yes	13	54
No	11	46
**Reported assumption or mechanism used in the analysis(*n*** **= 13)**		
MCAR	13	100
MAR	8	62
MNAR	9	75
MAR and MNAR	7	58
**The simulation method used before the analysis**		
Yes	4	17
No	20	83
**Details on the multiple Imputation method(*n*** **= 11)**		
Number of imputations stated	3	27
Variables included in the imputation model stated	7	64
**If the selection model used what was the selection variables? (*n*** **= 9)**		
Interviewer identity	9	100
Household visited on the first day of fieldwork	3	33
**If instrumental variable used what was the variables? (n = 2)**		
Interviewer identity	2	100
**Sensitivity analysis performed**		
Yes	6	25
No	18	75
**Changes reported to conclusion**		
No	2	8
Non-significant changes	11	46
Significant changes	11	46

## Discussion

We identified 69 citations that fulfilled our eligibility criteria on this HIV topic with only 24 studies addressing the missing data problem on the estimation of HIV prevalence during analysis. The same trend of fewer studies addressing the missing data problem is observed in other design like clinical trials and HIV longitudinal studies measuring different outcome [72]. The major reason for the missingness was reported to be a refusal to consent for an HIV test, and with complete case analysis be the primary method of analysis used. Multiple imputations and Heckman’s selection models were the major methods used to adjust for missing data, with 46% of studies showing a significant change of estimates after adjustments. Only a quarter of the included studies conducted a sensitivity analysis to assess the robustness of the results.

There was a good agreement between authors regarding the risk of bias, for all the included studies we had a high risk of bias on the domains assessing the internal validity of the studies compared to domains assessing the external validity, i.e. on the likelihood of non-participation. This may be because one criterion for the inclusion to the review was the study should have a line addressing the missing data or non-response problem.

The STROBE guideline [5] recommends that authors to report the amount of missing data, methods of handling missing data and the reasons for missingness s. However, of all included studies, only one was published before the STROBE guidelines in 2007, while others were published afterwards, and we found out that in most of the included studies provided the amount of missing data, with the corresponding reasons for missingness however, very few studies explored the differences between the participants with complete data and with missing data which can be used as the bases of examining the MCAR assumption.

The included studies used different methods for missing data analysis, and these ranged from ad hoc (complete case and single imputation) to advanced methods assuming MAR or MNAR mechanism (e.g., multiple imputations). Multiple imputations were the common method used despite that in most of the studies the methodology behind it was not clearly explained like the algorithm followed during imputation, number of imputed dataset and details on the imputation model. Provision of this information helps the replication of the methods and assessment of the results.

We observe an increase of the HIV prevalence estimates after adjusting for the missing data, demonstrating the presence of downward bias if complete case analysis is used The differences were significant in some studies [58, 71], and this suggests there might be underestimating of HIV prevalence if missing data are ignored.

All the applied methods had the shortcoming of its application considering the mechanism followed since there is no proof that missing data were MAR or MNAR. Heckman’s selection models and application of instrumental variables where the methods tried to explore the deviation of MAR to the possibility of MNAR assumption although a lack of suitable selection or instrumental variable impacts their applicability [57, 71]**.** The use of doubly robust methods and extension of Heckman’s selection models are the current methods identified as suitable when data are assumed to be MNAR. With the assumption that the missing data on HIV prevalence studies not being MAR, and the possibility of MNAR [54, 68], it is important to explore more methods than identified from this review.

Further to the analysis, a report from National Research Council (NRC) [73] explains the importance of conducting sensitivity analysis to assess the robustness of the results and conclusion of the assumptions used on the application of methods used to adjust for missing data. However, Only a quarter of the included studies performed a sensitivity analysis…. This does not differ with results provided by other reviews on missing dat, that very few studies assessed the robustness of the results regardless of the design [74, 75].

This is the first systematic review exploring the methods used in addressing the missing data problem on estimating HIV prevalence, however these results can only be generalizable to studies where the focus is on missing data This review will guide us in future application of these methods on real datasets from a population-based study conducted in North-West Tanzania and estimate the amount of bias caused by the missing data. Also, we will extend the methods assuming data being MNAR with further assessment by using a sensitivity analysis approach.

## Conclusion

This review aimed to look at surveys to determine what analytical methods or technique have been used to address the missing data problem on estimating HIV prevalence. From the studies included we saw that several methods can be used when data are not missing completely at random,. However, studies often report very little information on the steps, theories, assumptions and sensitivity of the reported results. .

All methods used for handling missing data in the included studies produced different estimates from the primary analysis, and in some studies, the difference was large. These differences highlight the need for considering using more advance methods when facing the problem of missing data in surveys and population studies to avoid producing biased results.

A further extension of this work is needed to compare the effectiveness of the estimates, and the amount of bias remaining from the available methods for dealing with missing data. Awareness is an important aspect of ensuring that these methods are applied appropriately, and the right choices are made considering the reasons, patterns and mechanism of the missing data..

## Supplementary information

**Additional file 1.** Search Strategy.

**Additional file 2.** Data extraction tool.

**Additional file 3.** Risk of Bias assessment domains.

**Additional file 4.** Risk of bias assessment table.

## Data Availability

Dataset used in the analysis will be made available from the corresponding author on reasonable request.

## Abbreviations

DHS: Demographic Health Surveys
MAR: Missing at Random
MCAR: Missing Completely at Random
MNAR: Missing Not at Random
NRC: National Research Council
STROBE: Strengthening the Reporting of Observational Studies in Epidemiology
