# Supplementary material for: Analytical methods used in estimating the prevalence of HIV/AIDS from demographic and cross-sectional surveys with missing data: a systematic review
Source: BMC Med Res Methodol. 2020 Mar 14;20:65. doi: 10.1186/s12874-020-00944-w (PMC7071763; doi:10.1186/s12874-020-00944-w)
Supplement: Supplementary file 1 — Additional file 1. Search Strategy. [file 12874_2020_944_MOESM1_ESM.docx]

**Appendix 1: Search Strategy**

**Medline (PubMed)**

| 1 | ((HIV OR hiv-1 OR hiv-2* OR hiv1 OR hiv2 OR hiv infect* OR human immunodeficiency virus OR human immune deficiency virus OR human immuno-deficiency virus OR human immune-deficiency virus OR ((human immun*) AND (deficiency virus)) OR acquired immunodeficiency syndromes OR acquired immune deficiency syndrome OR acquired immuno-deficiency syndrome OR acquired immune-deficiency syndrome OR ((acquired immun*) AND (deficiency syndrome)) OR HIV/AIDS)) |
| --- | --- |
| 2 | (HIV infections [MeSH] OR HIV [MeSH]) |
| 3 | (#1 OR #2) |
| 4 | (surveys or survey OR "Health Surveys"[Mesh] OR "Health Care Surveys"[Mesh] OR "Surveys and Questionnaires"[Mesh]) |
| 5 | (prevalence OR "Prevalence"[Mesh]) |
| 6 | (cross-sectional OR "cross sectional" OR crosssectional OR "Cross-Sectional Studies"[Mesh]) |
| 7 | (#4 OR #5 OR #6) |
| 8 | (missing) |
| 9 | (dropout* OR drop-out* OR "drop-out" OR "drop outs" OR attrition) |
| 10 | ("lost to followup" OR "lost to follow-up" OR "lost to follow up") |
| 11 | ("loss to followup" OR "loss to follow-up" OR "loss to follow up") |
| 12 | (#8 OR #9 OR #10 OR #11) |
| 13 | (#3 AND #7 AND #12) |
| 14 | (#13 AND Filters: Publication date from 2000/01/01) |

**Web of Science Core Collection (Clarivate Analytics)**

You searched for: TOPIC: (((((((((((((((((HIV OR hiv-1) OR hiv-2*) OR hiv1) OR hive) OR hiv infect*) OR human immunodeficiency virus) OR human immune deficiency virus) OR human immuno-deficiency virus) OR human immune-deficiency virus) OR (human immun* AND deficiency virus)) OR acquired immunodeficiency syndromes) OR acquired immune deficiency syndrome) OR acquired immuno-deficiency syndrome) OR acquired immune-deficiency syndrome) OR (acquired immun* AND deficiency syndrome)) OR HIV AIDS)) AND TOPIC: ((surveys or survey OR questionnaire OR questionnaires OR cross-sectional OR "cross sectional" OR crosssectional OR prevalence)) AND TOPIC: ((missing OR dropout* OR drop-out* OR "drop-out" OR "drop outs" OR attrition OR "lost to followup" OR "lost to follow-up" OR "lost to follow up" OR "loss to followup" OR "loss to follow-up" OR "loss to follow up"))

Timespan: 2000-2018. Indexes: SCI-EXPANDED, SSCI, CPCI-S.

**Latin American and Caribbean Health Sciences Literature (LILACS)**

Search on : (HIV$ OR HIV/AIDS OR AIDS OR immune-deficiency OR immunedeficiency OR immune deficiency OR immuno-deficiency OR immunodeficiency OR immuno deficiency) [Words] and (surveys or survey OR questionnaire OR questionnaires OR cross-sectional OR "cross sectional" OR crosssectional OR prevalence) [Words] and (missing OR dropout$ OR drop-out$ OR "drop-out" OR "drop outs" OR attrition OR "lost to followup" OR "lost to follow-up" OR "lost to follow up" OR "loss to followup" OR "loss to follow-up" OR "loss to follow up") [Words]

No date restriction

**Africa-Wide Information (EBSCObscohost)**

TX (HIV* OR HIV/AIDS OR AIDS OR immune-deficiency OR immunedeficiency OR immune deficiency OR immuno-deficiency OR immunodeficiency OR immuno deficiency)

AND

TX (surveys or survey OR questionnaire OR questionnaires OR cross-sectional OR "cross sectional" OR crosssectional OR prevalence)

AND

TX (missing OR dropout* OR drop-out* OR "drop-out" OR "drop outs" OR attrition OR "lost to followup" OR "lost to follow-up" OR "lost to follow up" OR "loss to followup" OR "loss to follow-up" OR "loss to follow up")

**Scopus**

( TITLE-ABS-KEY ( ( hiv* OR hiv/aids OR aids OR immune-deficiency OR immunedeficiency OR immune AND deficiency OR immuno-deficiency OR immunodeficiency OR immuno AND deficiency ) ) AND TITLE-ABS-KEY ( ( surveys OR survey OR questionnaire OR questionnaires OR cross-sectional OR "cross sectional" OR crosssectional OR prevalence ) ) AND TITLE-ABS-KEY ( ( missing OR dropout* OR drop-out* OR "drop-out" OR "drop outs" OR attrition OR "lost to followup" OR "lost to follow-up" OR "lost to follow up" OR "loss to followup" OR "loss to follow-up" OR "loss to follow up" ) ) ) AND PUBYEAR > 1999

Limiters: Year Published: 2000-2018
