# Supplementary material for: Analytical methods used in estimating the prevalence of HIV/AIDS from demographic and cross-sectional surveys with missing data: a systematic review
Source: BMC Med Res Methodol. 2020 Mar 14;20:65. doi: 10.1186/s12874-020-00944-w (PMC7071763; doi:10.1186/s12874-020-00944-w)
Supplement: Supplementary file 2 — Additional file 2. Data extraction tool. [file 12874_2020_944_MOESM2_ESM.docx]

**Appendix 2: Data extraction tool**

| **Title: Analytical methods used in handling missing data in estimating prevalence of HIV/AIDS for demographic and cross-sectional studies.** | | | | | | | | |  |
| --- | --- | --- | --- | --- | --- | --- | --- | --- | --- |
|  |  |  |  |  |  |  |  |  |  |
| **Part 1: Main survey** | | | | | | | |  |  |
|  | Study ID |  |  | | |  |  |  |  |
|  |  |  |  |  |  |  |  |  |  |
| 1 | Date of extraction: | |  | |  |  |  |  |  |
|  |  |  |  |  |  |  |  |  |  |
| 2 | Tittle of the article: | |  | | | | | |  |
|  |  |  |  |  |  |  |  |  |  |
| 3 | Name of the journal: | |  | | | | | |  |
|  |  |  |  |  |  |  |  |  |  |
| 5 | Year of publication: | |  | | | |  |  |  |
|  |  |  |  |  |  |  |  |  |  |
| 6 | Type of the study: | |  |  |  |  |  |  |  |
|  |  | 1 | Demographic/Household | | |  |  |  |  |
|  |  | 2 | Cross-sectional | |  |  |  |  |  |
|  |  | 3 | Population survey | |  |  |  |  |  |
|  |  |  |  |  |  |  |  |  |  |
| 7 | Place of the study: | |  | | |  |  |  |  |
|  |  |  |  |  |  |  |  |  |  |
| 8 | Year when the study was done | | |  | |  |  |  |  |
|  |  |  |  |  |  |  |  |  |  |
| 9 | Age of the participants: | | |  |  | | |  |  |
|  |  |  | Minimum | Maximum |  |  |  |  |  |
|  |  | Men |  |  |  |  |  |  |  |
|  |  | Women |  |  |  |  |  |  |  |
|  |  |  |  |  |  |  |  |  |  |
| 10 | What was the total number of individuals included in the study | | | | | |  |  |  |
|  |  |  |  |  |  |  |  |  |  |
|  |  |  |  |  |  |  |  |  |  |
| 11 | Response rate reported: | | |  |  |  |  |  |  |
|  |  | 0 | No |  |  |  |  |  |  |
|  |  | 1 | Yes |  |  |  |  |  |  |
|  |  | 2 | Not applicable | |  |  |  |  |  |
|  |  |  |  |  |  |  |  |  |  |
| 12 | If yes what was the reported response rate: | | |  |  |  |  |  |  |
|  |  |  | Overall |  |  |  |  |  |  |
|  |  |  | Males |  |  |  |  |  |  |
|  |  |  | Females |  |  |  |  |  |  |
|  |  |  |  |  |  |  |  |  |  |
| 13 | Sample size accounted for missing data: | | | |  |  |  |  |  |
|  |  | 0 | No |  |  |  |  |  |  |
|  |  | 1 | Yes |  |  |  |  |  |  |
|  |  | 2 | Not applicable | |  |  |  |  |  |
|  |  |  |  |  |  |  |  |  |  |
| 14 | Type of missing data | | |  |  |  |  |  |  |
|  |  | 1 | Unit non-response | |  |  |  |  |  |
|  |  | 2 | Item non-response | |  |  |  |  |  |
|  |  | 3 | Both types |  |  |  |  |  |  |
|  |  |  |  |  |  |  |  |  |  |
| 15 | The overall proportion of missing data mentioned in the study/ or per survey? | | | | | | |  |  |
|  |  | 0 | No |  |  |  |  |  |  |
|  |  | 1 | Yes |  |  |  |  |  |  |
|  |  |  |  |  |  |  |  |  |  |
| 16 | Proportion/Number of participants or variables with missing data mentioned | | | | | | | | |
|  |  | 0 | No |  |  |  |  |  |  |
|  |  | 1 | Yes |  |  |  |  |  |  |
|  |  |  |  |  |  |  |  |  |  |
| 17 | Reasons for missing data mentioned | | | |  |  |  |  |  |
|  |  | 0 | No |  |  |  |  |  |  |
|  |  | 1 | Yes |  |  |  |  |  |  |
|  |  |  |  |  |  |  |  |  |  |
| 18 | What were the mentioned reasons for missing data? | | | | |  |  |  |  |
|  |  | 1 |  | | | | |  |  |
|  |  | 2 |  | | | | |  |  |
|  |  | 3 |  | | | | |  |  |
|  |  | 4 |  | | | | |  |  |
|  |  | 5 |  | | | | |  |  |
|  |  |  |  |  |  |  |  |  |  |
| 19 | Was the missing data reported as a separate outcome? | | | | | |  |  |  |
|  |  | 0 | No |  |  |  |  |  |  |
|  |  | 1 | Yes |  |  |  |  |  |  |
|  |  |  |  |  |  |  |  |  |  |
| 20 | A summary table to assess the differences between the participants with complete and incomplete dataset provided? | | | | | |  |  |  |
|  |  | 0 | No |  |  |  |  |  |  |
|  |  | 1 | Yes |  |  |  |  |  |  |
|  |  |  |  |  |  |  |  |  |  |
| 21 | Statement regarding missing data pattern stated in the analysis | | | | | |  |  |  |
|  |  | 0 | No |  |  |  |  |  |  |
|  |  | 1 | Yes |  |  |  |  |  |  |
|  |  |  |  |  |  |  |  |  |  |
| 22 | Statement regarding missing data mechanism assumed in the analysis: | | | | | | |  |  |
|  |  | 0 | No |  |  |  |  |  |  |
|  |  | 1 | Yes |  |  |  |  |  |  |
|  |  |  |  |  |  |  |  |  |  |
| 23 | What type of missing data mechanism was assumed in the analysis: | | | | | | |  |  |
|  |  | 0 | Missing Completely at Random | | |  |  |  |  |
|  |  | 1 | Missing at Random | |  |  |  |  |  |
|  |  | 2 | Missing not at Random | | |  |  |  |  |
|  |  |  |  |  |  |  |  |  |  |
| 24 | The analytical method used to handle missing data | | | | | (Multiple responses) | |  |  |
|  |  | 1 | Complete case analysis | | |  |  |  |  |
|  |  | 2 | Last observation carried forward | | |  |  |  |  |
|  |  | 3 | Single imputation | |  |  |  |  |  |
|  |  | 4 | Multiple imputations | |  |  |  |  |  |
|  |  | 5 | Inverse probability weighting | | |  |  |  |  |
|  |  | 6 | Propensity scores | |  |  |  |  |  |
|  |  | 7 | Instrumental variables | | |  |  |  |  |
|  |  | 8 | Double robust methods | | |  |  |  |  |
|  |  | 9 | Maximum likelihood | |  |  |  |  |  |
|  |  | 10 | Heckman-type selection models | | |  |  |  |  |
|  |  | 11 | Other |  |  |  |  |  |  |
|  |  |  |  |  |  |  |  |  |  |
| 25 | If other method(s) mention: | | |  | | | |  |  |
|  |  | 1 |  | | | | |  |  |
|  |  | 2 |  | | | | |  |  |
|  |  | 3 |  | | | | |  |  |
|  |  |  |  |  |  |  |  |  |  |
| 26 | If selection models used, what were the selection variables | | | | | | |  |  |
|  |  | 1 |  | | | |  |  |  |
|  |  | 2 |  | | | |  |  |  |
|  |  | 3 |  | | | |  |  |  |
|  |  | 4 |  | | | |  |  |  |
|  |  |  |  |  |  |  |  |  |  |
| 27 | Adjustments made during analysis? | | | |  |  |  |  |  |
|  |  | 0 | No |  |  |  |  |  |  |
|  |  | 1 | Yes |  |  |  |  |  |  |
|  |  |  |  |  |  |  |  |  |  |
| 28 | The simulation method used prior application of the methods | | | | | |  |  |  |
|  |  | 0 | No |  |  |  |  |  |  |
|  |  | 1 | Yes |  |  |  |  |  |  |
|  |  | 2 | Unclear |  |  |  |  |  |  |
|  |  |  |  |  |  |  |  |  |  |
| 29 | The reason provided for a selected method(s) used for handling missing data used | | | | | | | | |
|  |  | 0 | No |  |  |  |  |  |  |
|  |  | 1 | Yes |  |  |  |  |  |  |
|  |  | 2 | Unclear |  |  |  |  |  |  |
|  |  |  |  |  |  |  |  |  |  |
| If multiple imputation used: | | | |  |  |  |  |  |  |
| 30 | Number of imputation and imputed datasets stated | | | | |  |  |  |  |
|  |  | 0 | No |  |  |  |  |  |  |
|  |  | 1 | Yes |  |  |  |  |  |  |
|  |  | 2 | Unclear |  |  |  |  |  |  |
|  |  |  |  |  |  |  |  |  |  |
| 31 | Variables included in the imputation model mentioned: | | | | | |  |  |  |
|  |  | 0 | No |  |  |  |  |  |  |
|  |  | 1 | Yes |  |  |  |  |  |  |
|  |  | 2 | Unclear |  |  |  |  |  |  |
|  |  |  |  |  |  |  |  |  |  |
|  |  |  |  |  |  |  |  |  |  |
| 32 | What where the estimates from the method used: | | | | |  |  |  |  |
|  | **Overall** | | | | | | |  |  |
|  |  | **n** | **Method used** | | | **Estimate** | **95% CI** | **SE** |  |
|  |  |  |  | | |  |  |  |  |
|  |  |  |  | | |  |  |  |  |
|  |  |  |  | | |  |  |  |  |
|  | **Males** | | | | | | |  |  |
|  |  |  |  | | |  |  |  |  |
|  |  |  |  | | |  |  |  |  |
|  |  |  | **Females** | | |  |  |  |  |
|  |  |  |  | | |  |  |  |  |
|  |  |  |  | | |  |  |  |  |
|  |  |  |  | | |  |  |  |  |
| 33 | What was the best-concluded method to use? | | | | |  | | |  |
|  |  |  |  |  |  |  |  |  |  |
|  |  |  |  |  |  |  |  |  |  |
| 34 | Sensitivity analysis performed after analysis | | | | |  |  |  |  |
|  |  | 0 | No |  |  |  |  |  |  |
|  |  | 1 | Yes |  |  |  |  |  |  |
|  |  |  |  |  |  |  |  |  |  |
| 35 | What approaches/methods were used in the sensitivity analysis | | | | | | |  |  |
|  |  | 1 |  | | | | |  |  |
|  |  | 2 |  | | | | |  |  |
