# Supplementary material for: Analytical methods used in estimating the prevalence of HIV/AIDS from demographic and cross-sectional surveys with missing data: a systematic review
Source: BMC Med Res Methodol. 2020 Mar 14;20:65. doi: 10.1186/s12874-020-00944-w (PMC7071763; doi:10.1186/s12874-020-00944-w)
Supplement: Supplementary file 3 — Additional file 3. Risk of Bias assessment domains. [file 12874_2020_944_MOESM3_ESM.docx]

**Appendix 3: Risk of Bias assessment domains**

| **No** | **Risk of Bias item** | **Level of Risk** | |
| --- | --- | --- | --- |
|  |  | **Yes (Low)** | **No (High)** |
|  | **External Validity** |  |  |
| D1 | Was the study target population a close representation of the general HIV population with relevant variables? |  |  |
| D2 | Was the sampling frame a true or close representation of the target population? |  |  |
| D3 | Was some form of random selection used to select the sample, OR, was a census undertaken? |  |  |
| D4 | Was the likelihood of non-participation bias minimal? |  |  |
|  | **Internal Validity** |  |  |
| D5 | Were data collected directly from the subjects (as opposed to medical records)? |  |  |
| D6 | Were an acceptable case definition of HIV/AIDS used? |  |  |
| D7 | Was a reliable and accepted diagnosis method for HIV/AIDS utilized? |  |  |
| D8 | Was the same mode of data collection used for all subjects? |  |  |
| D9 | Was the length of the shortest prevalence period for the parameter of interest appropiate? |  |  |
| D10 | Were the numerators(s) and denominators(s) for the calculation of the prevalence of HIV appropiate? |  |  |
| D11 | The summary item on the overall risk of study bias |  |  |
| **Overall agreement for the 11 items** | |  |  |
