# Supplementary material for: Analytical methods used in estimating the prevalence of HIV/AIDS from demographic and cross-sectional surveys with missing data: a systematic review
Source: BMC Med Res Methodol. 2020 Mar 14;20:65. doi: 10.1186/s12874-020-00944-w (PMC7071763; doi:10.1186/s12874-020-00944-w)
Supplement: Supplementary file 4 — Additional file 4. Risk of bias assessment table. [file 12874_2020_944_MOESM4_ESM.docx]

Appendix 4: Risk of bias assessment table

| **Study ID** | **Risk of Bias assessment domains** | | | | | | | | | | |
| --- | --- | --- | --- | --- | --- | --- | --- | --- | --- | --- | --- |
|  | **Internal** | | | | **External** | | | | | |  |
|  | **D1** | **D2** | **D3** | **D4** | **D5** | **D6** | **D7** | **D8** | **D9** | **D10** | **D11** |
| Barnighausen,2011 | Low | Low | Low | High | Low | Low | Low | Low | Low | Low | Low |
| Chinomona, 2015 | Low | Low | Low | High | Low | Low | Low | Low | Low | Low | Low |
| Clark,2014 | Low | Low | Low | High | Low | Low | Low | Low | Low | Low | Low |
| Floyd,2013 | High | Low | Low | High | Low | Low | Low | Low | Low | Low | Low |
| Harling,2017 | Low | Low | Low | High | Low | Low | Low | Low | Low | Low | Low |
| Hogan, 2012 | Low | Low | Low | High | Low | Low | Low | Low | Low | Low | Low |
| Jessens,2014 | High | Low | Low | High | Low | Low | Low | Low | Low | Low | Low |
| Kendall,2014 | High | Low | High | Low | Low | Low | Low | Low | Low | Low | High |
| Kerr,2013 | High | Low | High | High | Low | Low | Low | Low | Low | Low | High |
| Kerr,2018 | High | Low | High | High | Low | Low | Low | Low | Low | Low | High |
| Leacy,2017 | High | Low | Low | High | Low | Low | Low | Low | Low | Low | Low |
| Mara,2017 | Low | Low | Low | High | Low | Low | Low | Low | Low | Low | Low |
| Marden,2018 | Low | Low | Low | High | Low | Low | Low | Low | Low | Low | Low |
| Marino,2018 | Low | Low | Low | High | Low | Low | Low | Low | Low | Low | Low |
| Marston,2008 | Low | Low | Low | High | Low | Low | Low | Low | Low | Low | Low |
| McGovern,2015a | High | Low | Low | High | Low | Low | Low | Low | Low | Low | Low |
| McGovern,2015b | Low | Low | Low | High | Low | Low | Low | Low | Low | Low | Low |
| McGovern,2015c | Low | Low | Low | High | Low | Low | Low | Low | Low | Low | Low |
| Mishra, 2006 | Low | Low | Low | High | Low | Low | Low | Low | Low | Low | Low |
| Mishra, 2008 | Low | Low | Low | High | Low | Low | Low | Low | Low | Low | Low |
| Reniers, 2009a | High | High | High | High | Low | Low | Low | Low | Low | Low | High |
| Reniers, 2009b | Low | Low | Low | High | Low | Low | Low | Low | Low | Low | Low |
| Tchetgen, 2013 | High | Low | Low | High | Low | Low | Low | Low | Low | Low | Low |
| Ziraba,2010 | High | Low | Low | High | Low | Low | Low | High | Low | Low | Low |
